# The polish wheat (*Triticum polonicum* L.) *TpSnRK2.10* and *TpSnRK2.11* meditate the accumulation and the distribution of cd and Fe in transgenic *Arabidopsis* plants

**DOI:** 10.1186/s12864-019-5589-1

**Published:** 2019-03-12

**Authors:** Ruijiao Wang, Chao Wang, Qin Yao, Xue Xiao, Xing Fan, Lina Sha, Jian Zeng, Houyang Kang, Haiqin Zhang, Yonghong Zhou, Yi Wang

**Affiliations:** 1 Triticeae Research Institute, Sichuan Agricultural University, Wenjiang, 611130 Sichuan China; 2 Joint International Research Laboratory of Crop Resources and Genetic Improvement, Sichuan Agricultural University, Wenjiang, 611130 Sichuan China; 3 College of Resources, Sichuan Agricultural University, Wenjiang, 611130 Sichuan China

**Keywords:** Wheat, TpSnRK2, Cd, Biological function, Expression pattern

## Abstract

**Background:**

The SnRK2s (Plant specific protein kinase) are involved in various biological processes, such as plant defense and environmental challenges. In *Arabidopsis*, AtSnRK2s regulate the expression of some metal transporters. For example, AtSnRK2.4 plays a role in the regulation of *Arabidopsis* tolerance to Cd; AtSnRK2.2 and AtSnRK2.3 are involved in Cd uptake and translocation. However, the functions of their homologs, *TpSnRK2.10* and *TpSnRK2.11* from dwarf Polish wheat are unknown.

**Results:**

*TpSnRK2.11* encodes a cytoplasmic protein. *TpSnRK2.10* and *TpSnRK2.11* have different expression patterns at different growth stages. Expression of *TpSnRK2.10* increased yeast’s sensitivity to Cd; conversely, expression of *TpSnRK2.11* enhanced yeast’s tolerance to Cd. Overexpression of *TpSnRK2.10* or *TpSnRK2.11* did not affect Cd sensitivity in *Arabidopsis*, but significantly increased Cd accumulation in roots and shoots, and Cd translocation from roots to shoots. While, Fe accumulation was significantly increased in roots but decreased in shoots by overexpression of *TpSnRK2.10*; opposite results were observed in *TpSnRK2.11-*overexpressing lines. Subcellular distribution analysis found that overexpression of *TpSnRK2.10* and *TpSnRK2.11* increased Cd concentration in cell wall and organelle fractions of roots and shoots; meanwhile, they also differentially influenced Fe distribution.

**Conclusions:**

These results indicated that TpSnRK2.10 and TpSnRK2.11 are involved in the uptakes and the translocations of Cd and Fe, possibly by regulating the expression of *AtNRAMP1* and *AtHMA4*, and other genes involved in the synthesis of phytochelatins or hemicellolosic polysaccharides.

**Electronic supplementary material:**

The online version of this article (10.1186/s12864-019-5589-1) contains supplementary material, which is available to authorized users.

## Background

More arable land soils have been contaminated by cadmium (Cd) through various industrial processes and/or agricultural practices [1]. As one of the toxic heavy metals, Cd is readily taken up by plants, and then entries into food chain, which potentially affects human health [2]. In plants, Cd is absorbed via basal roots, loaded in the xylem, then transported to shoots [3, 4], which negatively affects mineral nutrition and homeostasis in tissues and root growth and development [2, 5–7]. Characterization of numerous Cd transporters responsible for Cd accumulation and transport found that they are regulated by intricate stress signal transduction pathways [2, 5]. Thus, our knowledge about Cd accumulation and transport remains limited; the investigation is still needed to understand the molecular mechanisms.

The Cd signal transduction pathway is mediated by plant protein kinases that are major signal transduction elements [8, 9]. For example, Cd stress induces the activities of mitogen-activated protein kinases and sucrose nonfermenting-1 (SNF1)-related protein kinase 2 (SnRK2) in plants [6, 8, 9]. As plant specific protein kinase, ten SnRK2s are individually identified from *Arabidopsis*. Molecular analysis of them indicated that they are involved in various biological processes, such as plant defense to biotic and/or abiotic challenges [6, 10–12]. *AtSnRK2.2* and *AtSnRK2.3* regulate abscisic acid (ABA) response element (ABRE)-driven gene expression through the phosphorylation of ABRE binding factors (ABFs) [10]. Knockout of *AtSnRK2.2* and *AtSnRK2.3* enhances the expression of *IRON-REGULATED TRANSPORTER1* (*IRT1*) under 10 μM Cd stress [12]. IRT1 is a key transporter responsible for Cd uptake [3, 13]. The knockout of *IRT1* reduced Cd concentration in roots and increases in shoots [12], which is not consistent with its enhanced expression. Thus, knockout of *AtSnRK2.2* and *AtSnRK2.3* may regulate other metal transporters responsible for Cd uptake and translocation, such as *AtNRAMPs* whose promoters have an ABF or ABFs in *Arabidopsis* [14, 15]. In contrast, although knockout of *AtSnRK2.4* did not affect the expression of *IRT1* and the concentration of Cd under 20 μM Cd stress, it enhanced Cd tolerance and reduced phytochelatins concentration [6]. Phytochelatins chelate Cd to form phytochelatin-Cd complexes, which are transported into vacuoles to enhance Cd tolerance [16]. Meanwhile, AtSnRK2.6 probably participates in multiple signaling pathways. Inactivation of AtSnRK2.6 impairs stomatal close to enhance transpiration [17]. Overexpression of *AtSnRK2.6* increases the content of fructose and glucose [18]. Hemicellolosic polysaccharides of cell wall, consisted of fructose, glucose and other sugars, are major Cd binding sites, and positively correlate with the acquire capacity of Cd [19]. These results indicate that SnRK2s could be involved in Cd uptake and translocation; meanwhile, their mechanisms and functions may be different.

Several SnRK2s from rice and wheat, including OsSAPK8, OsSAPK9, OsSAPK10 and TaPKABA1, can also phosphorylate ABFs [20, 21]. Thus, they also potentially regulate the expression of genes with an ABRE or ABREs in their promoters. In wheat, 10 *SnRK2s* (*TaSnRK2.1*-*TaSnRK2.10*) are isolated and grouped into three subclasses. Subclass III *TaSnRK2s* are regulated by ABA stress, suggesting that these genes are involved in ABA regulated stress responses; however, subclass I and II *TaSnRK2s* are regulated by PEG, NaCl and cold stress, but not by ABA stress, suggesting that these genes responded to various abiotic stressors in an ABA-independent manner [22]. In tetraploid wheat, eight *SnRK2s* (*TpSnRK2.1*, *2.2*, *2.3*, *2.5*, *2.7*, *2.8*, *2.10* and *2.11*) are cloned from Polish wheat (*Triticum polonicum* L.); except of *TpSnRK2.7*, all of the genes are differentially regulated by ABA, NaCl, PEG and cold stress [23, 24]. However, whether these genes involved in Cd uptake and translocation are still unknown. Since *TpSnRK2.10* is homologous with *AtSnRK2.2* and *AtSnRK2.3*, and *TpSnRK2.11* is homologous with *AtSnRK2.4*, we hypothesized that they are involved in Cd uptake, translocation and tolerance. To test these hypotheses, we investigated their biological functions by analyzing expression pattern, subcellular localization in *Arabidopsis* leaf protoplast, Cd tolerance in yeast, and the concentration and subcellular distribution of Cd, Fe and Zn in *Arabidopsis* overexpressing lines.

## Methods

### Materials

Dwarf Polish wheat (*Triticum polonicum* L., 2n = 4X = 28, AABB, DPW) used in the present study was collected from Tulufan of Xinjiang province, China. Previous study indicated that the seedling of DPW exhibits high tolerance to Cd [25]. Thus, it is a desirable material for analyzing the mechanism of high tolerance to Cd. The seed of *Arabidopsis thaliana* (wild type, WT) was provided by Professor Yan Huang (College of Life Science, Sichuan Agricultural University).

### Bioinformatics and phylogenetic analysis

The full length cDNAs of *TpSnRK2.10* (KF688099) and *TpSnRK2.11* (KF688100) amplified from DPW were described by Wang et al. [23]. Their gene structures and chromosome localizations were analyzed using BLAST to search against the genome of *Triticum aestivum* [26] on website of Ensemblplants (http://plants.ensembl.org/Triticum_aestivum/Tools/Blast?db=core). Their putative subcellular localizations were predicted by the ProtComp 9.0 (http://linux1.softberry.com/berry.phtml?group=programs&subgroup=proloc&topic=protcomppl). The software of Vector NTI 11.5.1 (Invitrogen, Carlsbad, CA, USA) was used to align amino acid sequences including TpSnRK2.10, TpSnRK2.11, AtSnRK2.2, AtSnRK2.3 and AtSnRK2.4.

### Expression analysis of *TpSnRK2.10* and *TpSnRK2.11*

For expression pattern in DPW grown in normal growth season, tissues were collected from three growth stages with three biological replicates, including jointing stage (root, basal stem, leaf sheath, new leaf and young leaf), booting stage (root, basal stem, second stem, first node, second node, third node, lower leaf, flag leaf sheath, flag leaf blade and peduncle), and grain filling stages (root, stem, first node, leaf sheath, flag leaf blade, lemma and grain). All tissues were snap frozen in liquid nitrogen and stored at − 80 °C for RNA extraction.

For response to Cd stress and Fe-deficient in DPW seedlings, sterilized DPW seeds were germinated in dark at room temperature for four days. Uniform seedlings were cultured in Hoagland’s nutrient solution in a growth chamber for two weeks. All seedlings were treated with two stresses including 80 μM CdCl_2_ (Cd), and Fe-free Hoagland’s nutrient solution (Fe-deficient). Seedlings grown in Normal Hoagland’s nutrient solution were used as control (CK). After a week of treatments, roots were collected with three biological replicates and snap frozen in liquid nitrogen for RNA extraction.

Total RNA of each sample was isolated using the Total RNA Kit II (Omega, USA) according to the user manual. RNA concentration was measured by NanoDrop ND-1000 spectrophotometer (NanoDrop Technologies). The first-strand cDNA was synthesized from 2 μg total RNA using the M-MLV First Strand cDNA Synthesis Kit (Omega, USA).

Relative expression of *TpSnRK2.10* and *TpSnRK2.11* was normalized as described by Wang et al. [23] that included the information of *TpSnRK2.10-* and *TpSnRK2.11*-specific primers, and reference gene (*actin*).

### Subcellular localization of TpSnRK2.11 in *Arabidopsis* protoplasts

Subcellular localization of TpSnRK2.11 was analyzed as described by Wang et al. [4] and Peng et al. [27]. Briefly, the open reading frame of *TpSnRK2.11* was sub-cloned into the *KpnI* and *XbaI* sites of the *Arabidopsis* protoplast expression vector HBT95-GFP using *TpSnRK2.11* primers (F:CGGGGTACCATGGACAAGT ACGAGGAGG, and R: GCTCTAGATTAGATGTGCAACGCGCTCA). *Arabidopsis* leaf mesophyll protoplast was prepared and transformed according to the method of Yoo et al. [28]. Signal of GFP was detected by a confocal laser scanning microscope (Olympus, Tokyo, Japan).

### Cd sensitive analysis in yeast

Cd sensitivity in yeast was tested as described by Peng et al. [27]. Briefly, the open reading frames of *TpSnRK2.10* and *TpSnRK2.11* were individually sub-cloned into the *KpnI* and *XbaI* sites of yeast expression vector pYES2 using *TpSnRK2.11* primers and *TpSnRK2.10* primers (F: CGGGGTACCATGGACCGGGCGGCGC, and R: TGCTC TAGATCACATAGCATACAC). The S. C. EasyComp Transformation kit (Invitrogen, USA) was used to transform plasmid into yeast strains including wild type (BY4743) and Cd sensitive strain (ydr135c) according to its user manual.

For Cd sensitive testing on synthetic defined (SD) plate medium, positive cell suspension with optical density OD_600_ = 0.6 was diluted to four sequential dilutions (1:10, 1:100, 1:1000, and 1:10000). Diluted cell suspensions (5 μL) were cultured on SD plate medium containing 2% glucose or galactose, and CdCl_2_ (0 μM, 80 μM, and 100 μM), at 30 °C for 4 days.

For Cd sensitive testing in SD liquid medium, diluted cell suspensions (5 μL, 1:10000) were cultured on SD liquid medium containing 2% galactose and CdCl_2_ (0 μM and 80 μM), at 30 °C with shaking at 250 rpm. OD_600_ values were investigated at 0, 2, 4, 6, 8, 16, 24, 32, 40 and 48 h using a NanoDrop ND-1000 spectrophotometer (NanoDrop Technologies). All values were measured with three biological replicates.

### Functional analysis of *TpSnRK2.10* and *TpSnRK2.11* in overexpressing *Arabidopsis* lines

Biological functions of *TpSnRK2.10* and *TpSnRK2.11* were analyzed in *Arabidopsis* overexpressing lines as described by Peng et al. [27]. Briefly, the open reading frames of *TpSnRK2.10* and *TpSnRK2.11* were sub-cloned into the *KpnI* and *XbaI* sites of the expression vector pCAMBIA1305.1 driven by the 35S promoter. *TpSnRK2.10* and *TpSnRK2.11* were individually transformed into WT *Arabidopsis thaliana* plants using floral infiltration via *Agrobacterium*-mediated transformation [29] (two time-independent transformations). Homozygous overexpressing lines were selected using hygromycin selection and PCR with gene-specific primers [23]. The relative expression of *TpSnRK2.10* and *TpSnRK2.11* in their individually transgenic lines was analyzed as described as the section of “Expression analysis of *TpSnRK2.10* and *TpSnRK2.11*”. Meanwhile, the relative expression of *AtIRT1*, *AtNRAMP1* and *AtHMA4* was also investigated in WT, vector line, *TpSnRK2.10*- and *TpSnRK2.11*-expressing lines using *AtActin2* as reference gene. The qPCR primers of *AtIRT1*, *AtNRAMP1*, *AtHMA4* and *AtActin2* were used as described by Ihnatowicz et al. [30], Boonyaves et al. [31] and Chen et al. [32], respectively.

For investigation of Cd sensitive in *TpSnRK2.10*- and *TpSnRK1.11*-expressing lines, sterilized seeds of WT, an empty vector line and independent homozygous lines (each line contained three plant lines selected from an independent transformation; totally six plant lines) were germinated on 1/2 MS solid plates containing CdCl_2_ (0 μM and 40 μM) in a light incubator with 120 μE m^− 2^ s^− 1^ illumination intensity, 16/8 h light/dark period, 22 °C temperature and 50% humidity. On the tenth day, root length of each line was measured.

To investigate whether TpSnRK2.10 and /or TpSnRK2.11 influenced metal (Cd, Fe and Zn) concentration in *TpSnRK2.10*- and *TpSnRK1.11*-overexpressing lines, uniform seedlings of WT, an empty vector line and independent homozygous lines were cultured in soil. After three weeks, soil was added once with CdCl_2_ (0 mg/kg and 40 mg/kg) dissolved in water. After four weeks, dry weight of each plant was measured. Part of roots and shoots was dried at 80 °C for two days to measure metal concentration as described by Cheng et al. [7]. Remaining roots and shoots were used to measure the subcellular distribution of Cd and Fe as described by Cheng et al. [7].

### Data analysis

All data was statistically analyzed using Tukey’s test at *P* ≤ 0.05 in SPSS 20.0. Figures were drawn by the software of SigmaPlot 12.0.

## Results

### Chromosome localizations and gene structures of *TpSnRK2.10* and *TpSnRK2.11*

Blast search of the open reading frames of *TpSnRK2.10* and *TpSnRK2.11* against the genome of *T. aestivum* revealed that *TpSnRK2.10* is located on chromosome 4AL (gene: TRIAE_CS42_4AL_TGACv1_291776_AA0996560) and comprises eight exons and seven introns; *TpSnRK2.11* is located on chromosome 1BL (gene: TRIAE_CS42_1BL_TGACv1_031102_AA0107780) and comprises nine exons and eight introns.

### Homology analysis of TpSnRK2.10 and TpSnRK2.11 with AtSnRK2.2, AtSnRK2.3 and AtSnRK2.4

**Fig. 1:**
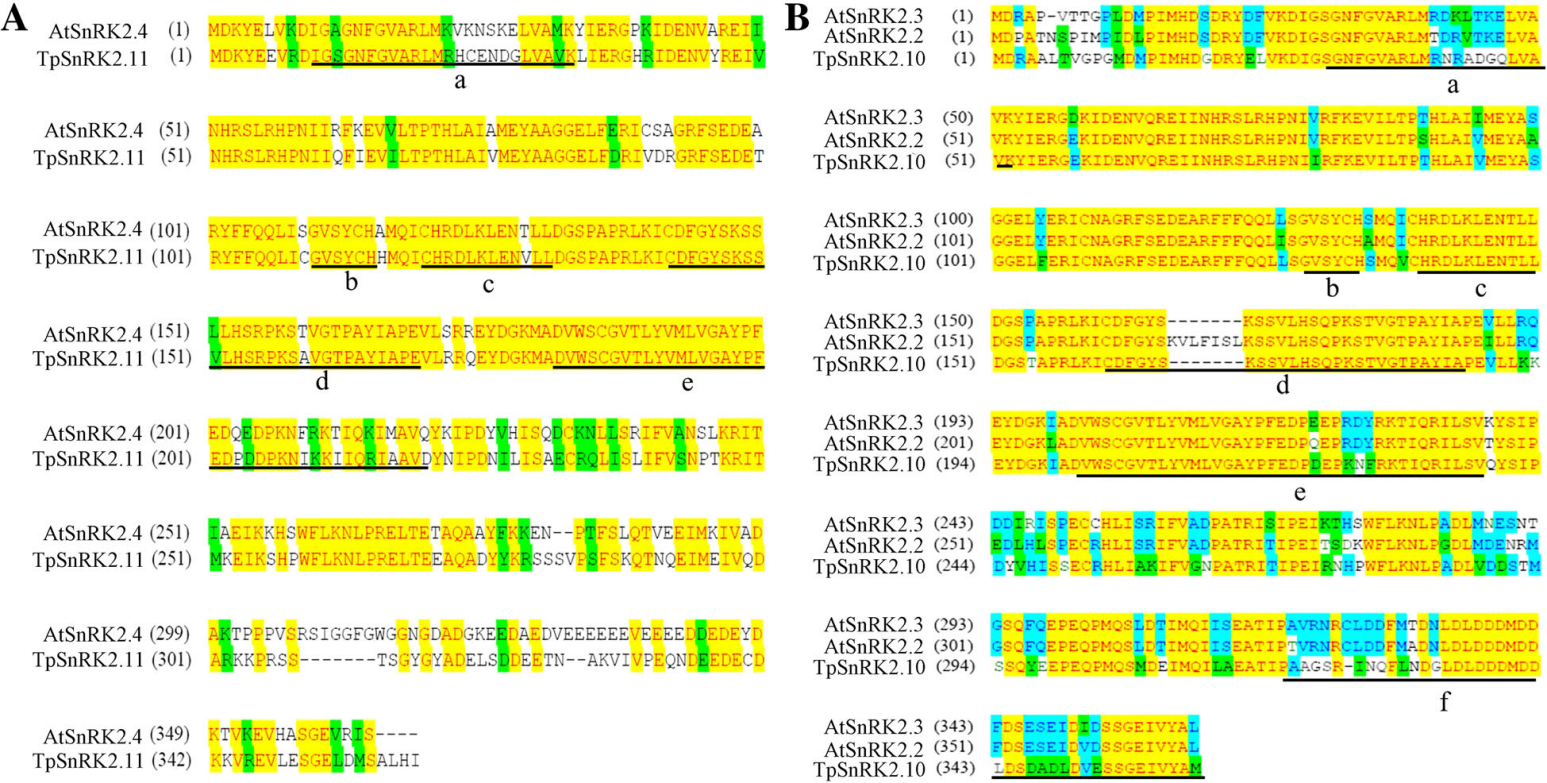
Alignments of TpSnRK2.10, AtSnRK2.2 and AtSnRK2.3, and TpSnRK2.11 and AtSnRK2.4. **a**: alignment of the amino acid sequences of TpSnRK2.11 and AtSnRK2.4; **b**: alignment of the amino acid sequences of TpSnRK2.10, AtSnRK2.2 and AtSnRK2.3. **a**: ATP binding region; **b**: N-myristoylation site; c: serine/threonine protein kinase activity site; d: activation loop; e: transmembrane helix site; f: required for ABA response

Previously phylogenetic analysis revealed that TpSnRK2.10 grouped with AtSnRK2.2 and AtSnRK2.3, which belong to the subgroup 3 kinases that are strongly activated by ABA; TpSnRK2.11 grouped with AtSnRK2.4, which belongs to the subgroup 1 kinases that are not activated by ABA. Alignment found that TpSnRK2.11 had a 68.6% identity to AtSnRK2.4 (Fig. 1a); TpSnRK2.10 had a 76.7 and 80.7% identity to AtSnRK2.2 and AtSnRK2.3, respectively (Fig. 1b). Further analysis revealed that except for N-myristoylation site, other protein kinases motifs (including ATP-binding region, serine/threonine protein kinases activity site, activation loop, and transmembrane helix site) had one and/or more than one residue differences between TpSnRK2.11 and AtSnRK2.4 (Fig. 1a). While, only three protein kinases motifs (including ATP-binding region, transmembrane helix site, and required for ABA responses site) had residue differences among TpSnRK2.10, AtSnRK2.2 and AtSnRK2.3 (Fig. 1b). These residue differences in motifs implied functional divergence between TpSnRK2.11 and AtSnRK2.4, and among TpSnRK2.10, AtSnRK2.2 and AtSnRK2.3.

### Expression patterns of *TpSnRK2.10* and *TpSnRK2.11*

**Fig. 2:**
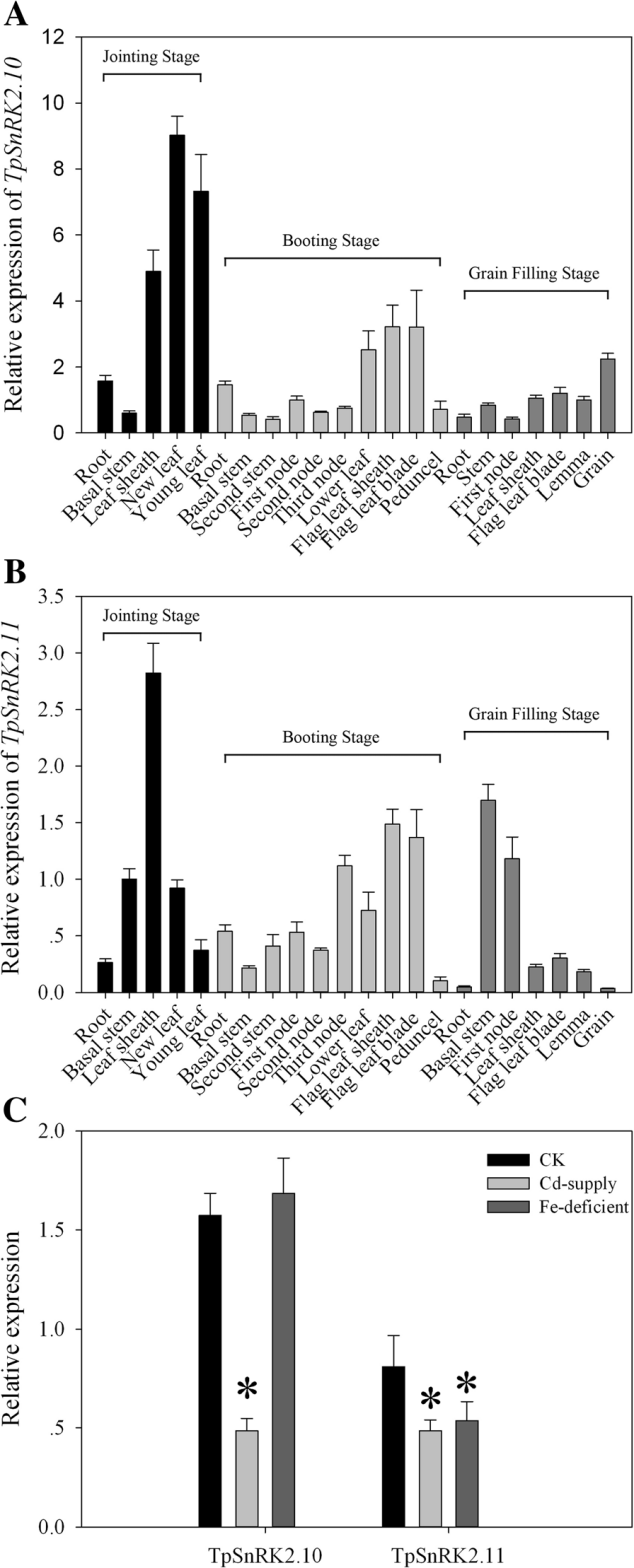
Expression patterns of *TpSnRK2.10* and *TpSnRK2.11*. **a** Expression level of *TpSnRK2.10* in different tissues at three growth stages; **b** expression level of *TpSnRK2.11* in different tissues at three growth stages; **c** expression levels of TpSnRK2.10 and TpSnRK2.11 under Cd-supply and Fe-deficient stresses. Jointing stage: the basal stem emerges above the soil line; booting stage: the developing head with the sheath of the flag leaf becomes visibly enlarged. Asterisk indicated significant difference when compared with WT at *P* < 0.05; values were means ± standard deviation (three biological replicates)

Expression patterns of *TpSnRK2.10* and *TpSnRK2.11* were investigated in different tissues at jointing, booting and grain-filling stages of wheat grown in a field. At jointing stage, expression of *TpSnRK2.10* was the highest in new leaves and young leaves, followed by leaf sheathes, and the lowest in basal stems (Fig. 2a). At booting stage, *TpSnRK2.10* was expressed the highest in lower leaves, flag leaf sheathes and flag leaf blades, and the lowest in basal stems and second stems (Fig. 2a). At grain-filling stage, the highest expression of *TpSnRK2.10* was observed in grains, and the lowest was found in roots and first nodes (Fig. 2a).

The expression pattern of *TpSnRK2.11* was different from that of *TpSnRK2.10* (Fig. 2b). At jointing stage, the highest expression of *TpSnRK2.11* was found in leaf sheathes, followed by in basal stems and new leaves, and the lowest was in roots and young leaves (Fig. 2b). At booting stage, *TpSnRK2.11* was mainly expressed in flag leaf sheathes, flag leaf blades and third nodes, then in lower leaves, roots and first nodes, and the lowest in basal stems and peduncels (Fig. 2b). At grain-filling stage, expression of *TpSnRK2.11* was the highest in basal stems and first nodes, and the lowest in roots and grains (Fig. 2b).

Meanwhile, the responses to Cd-supply and Fe-deficient were investigated (Fig. 2c). The expressions of *TpSnRK2.10* and *TpSnRK2.11* were dramatically down-regulated by Cd stress when compared with their individually CK (Fig. 2c). The expression of *TpSnRK2.10* was not regulated by Fe-deficient; while the expression of *TpSnRK2.11* was down-regulated by Fe-deficient (Fig. 2c). These results indicate that TpSnRK2.10 and TpSnRK2.11 are response to Cd and Fe.

### Subcellular localization of TpSnRK2.11

**Fig. 3:**
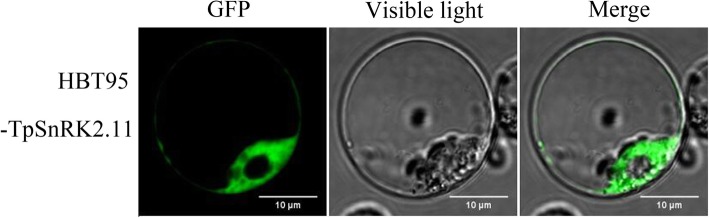
Subcellular localization of TpSnRK2.11

TpSnRK2.10 and TpSnRK2.11 were predicated to localize to the cytoplasm and the nucleus. To confirm the predicated subcellular localizations, a HBT95-*TpSnRK2.11*-*GFP* fusion vector was transiently transformed into *Arabidopsis* leaf protoplasts. Our previous study indicated that green fluorescence of the empty vector HBT95-GFP is located to the cytoplasm, the nucleus and the plasma membrane [4]. In this study, the green fluorescence of HBT95-TpSnRK2.11-GFP in *Arabidopsis* leaf protoplasts was detected in the cytoplasm, but was not in the nucleus (Fig. 3). Thus, *TpSnRK2.11* encodes a cytoplasmic protein. Unfortunately, we failed to detect the subcellular localization of TpSnRK2.10.

### Cd sensitivity in yeast

**Fig. 4:**
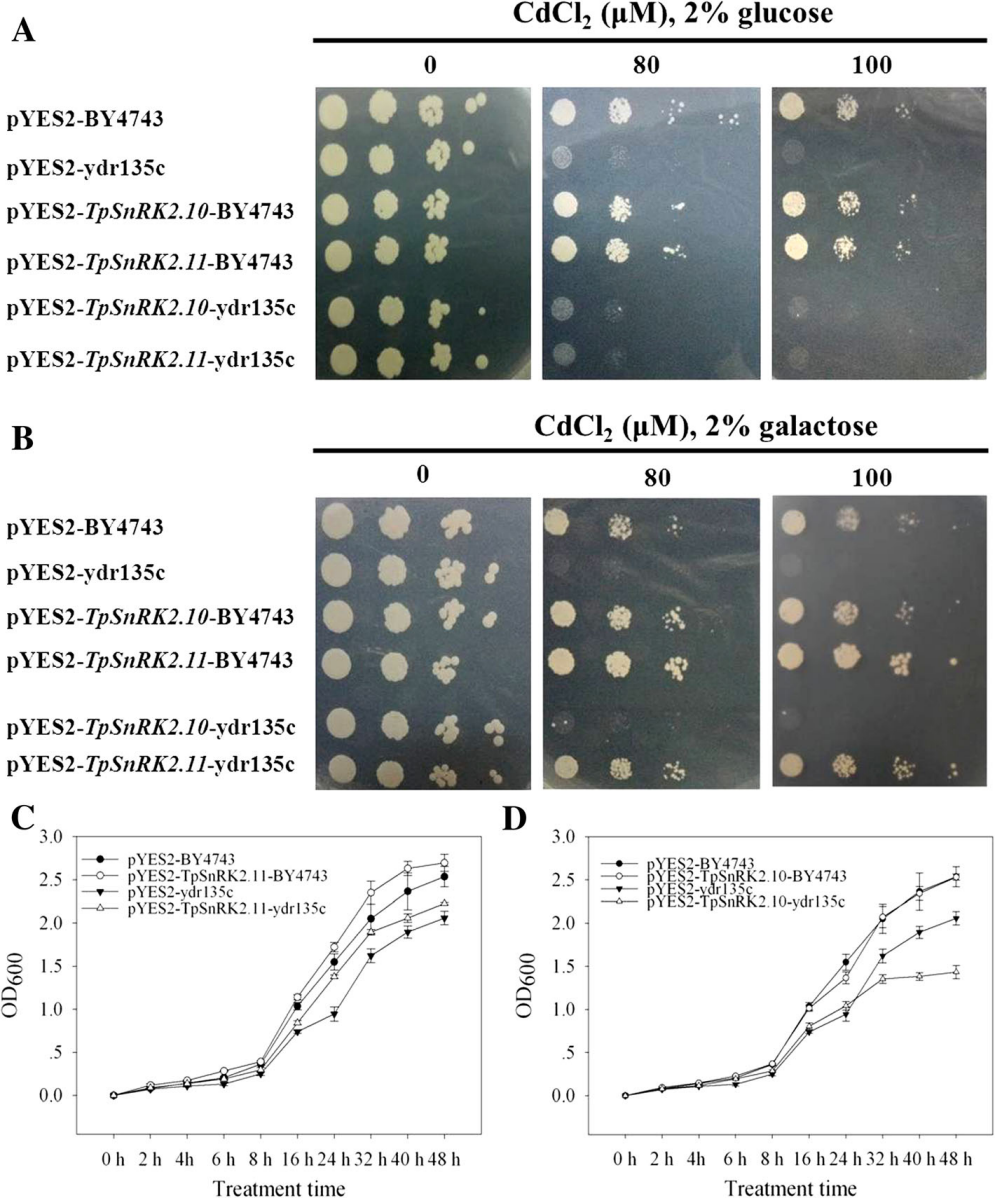
Sensitivity to Cd in yeast. **a-b** Sensitivity to Cd when grown on plate SD medium with 0, 80 or 100 μM CdCl_2_ under 2% glucose (A) or 2% galactose (B), at 30 °C for 4 days; serial 1:10 dilutions were shown from left to right for yeast wild type (BY4743) and Cd sensitive strain (ydr135c) transformed with empty vector (pYES2), *TpSnRK2.10* or *TpSnRK2.11*; **c-d** sensitivity to Cd when grown in liquid SD medium with 80 μM CdCl_2_ under 2% galactose; diluted cell suspensions (5 μL, 1:10000) were cultured from 0 to 48 h

To investigate whether TpSnRK2.10 and TpSnRK2.11 alter Cd sensitivity in yeast, we expressed *TpSnRK2.10*, *TpSnRK2.11* or pYES2 in BY4743 and ydr135c. In the presence of 2% glucose that represses gene expression with the *GAL1* promoter in pYES2 vector [33, 34], Cd stress significantly inhibited the growth of ydr135c when compared with BY4743 (Fig. 4a); meanwhile, similar growths were detected among ydr135c individually transformed with pYES2, *TpSnRK2.10* and *TpSnRK2.11* (Fig. 4a). In the presence of 2% galactose that induces gene expression with the *GAL1* promoter in pYES2 vector, expression of *TpSnRK2.11* dramatically increased the growth of ydr135c when compared with ydr135c transformed with pYES2 under both of 80 μM and 100 μM CdCl_2_ stresses (Fig. 4b); while expression of *TpSnRK2.10* did not change the growth of ydr135c when compared with ydr135c transformed with pYES2 under CdCl_2_ stresses (Fig. 4b).

To confirm the results produced from plate testing, the growth curves of transformed yeast grown in liquid medium with 80 μM CdCl_2_ were investigated (Fig. 4c and d). Compared with ydr135c transformed with pYES2, expression of *TpSnRK2.11* significantly increased the OD_600_ values of ydr135c starting at 16 h (Fig. 4c); while, expression of *TpSnRK2.10* dramatically reduced the OD_600_ values of ydr135c staring at 32 h (Fig. 4d). These results indicated that expression of *TpSnRK2.11* enhances Cd tolerance in yeast; while, expression of *TpSnRK2.10* increases Cd sensitivity in yeast.

### Functional overexpression of *TpSnRK2.10* and *TpSnRK2.11* in *Arabidopsis*

**Fig. 5:**
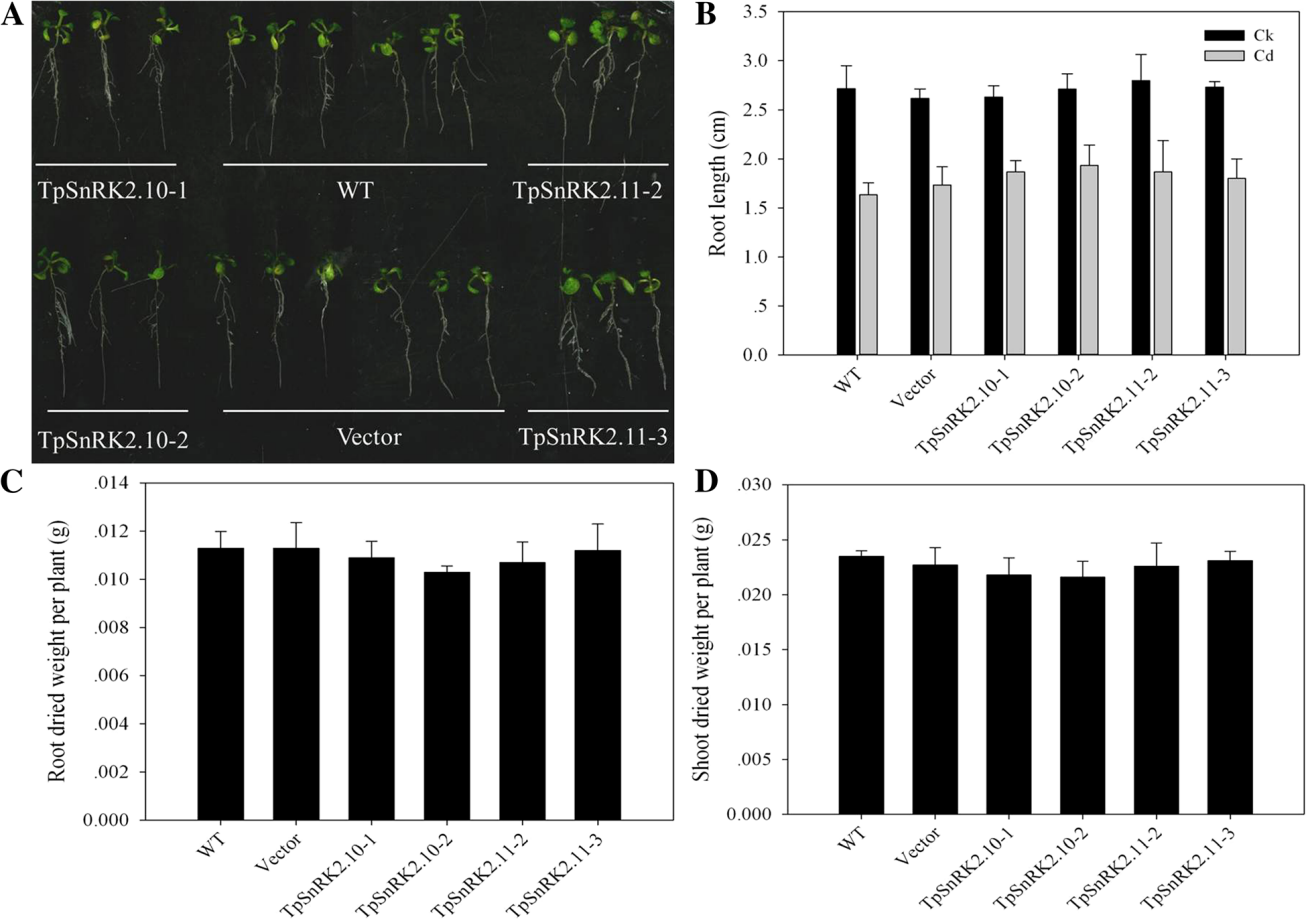
Phenotypes of *TpSnRK2.10*- and *TpSnRK2.11*-overexpressing *Arabidopsis* grown in SD plate with 40 μM CdCl_2_ or in soil with 40 mg/kg CdCl_2_. **a-b** Root lengths of *TpSnRK2.10*- and *TpSnRK2.11*-overexpressing *Arabidopsis* grown in SD plate with 40 μM CdCl_2_; **c-d** root (C) and shoot (D) dried weight per plant of *TpSnRK2.10*- and *TpSnRK2.11*-overexpressing *Arabidopsis* grown in soil with 40 mg/kg CdCl_2_

Since expression of *TpSnRK2.10* and *TpSnRK2.11* altered Cd sensitivity in yeast, it is interesting to investigate whether they would play roles in metal transport in plant. Thus, we individually overexpressed *TpSnRK2.10* and *TpSnRK2.11* in *Arabidopsis*. Two independent homozygous of *TpSnRK2.10*- or *TpSnRK2.11*-overexpressing lines were developed (Additional file 1: Figure S1). When grown on 1/2 MS medium with 40 μM CdCl_2_, overexpression of *TpSnRK2.10* or *TpSnRK2.11* did not change root length compared with WT (Fig. 5a and b); Cd stress significantly inhibited root length when compared with CK (Fig. 5b). When grown in soil with 40 mg/kg CdCl_2_, overexpression of *TpSnRK2.10* or *TpSnRK2.11* also did not change the dried weight of roots and shoots compared with CK (Fig. 5c and d). These results indicated that overexpression of *TpSnRK2.10* or *TpSnRK2.11* did not influence Cd sensitivity in plant.

**Fig. 6:**
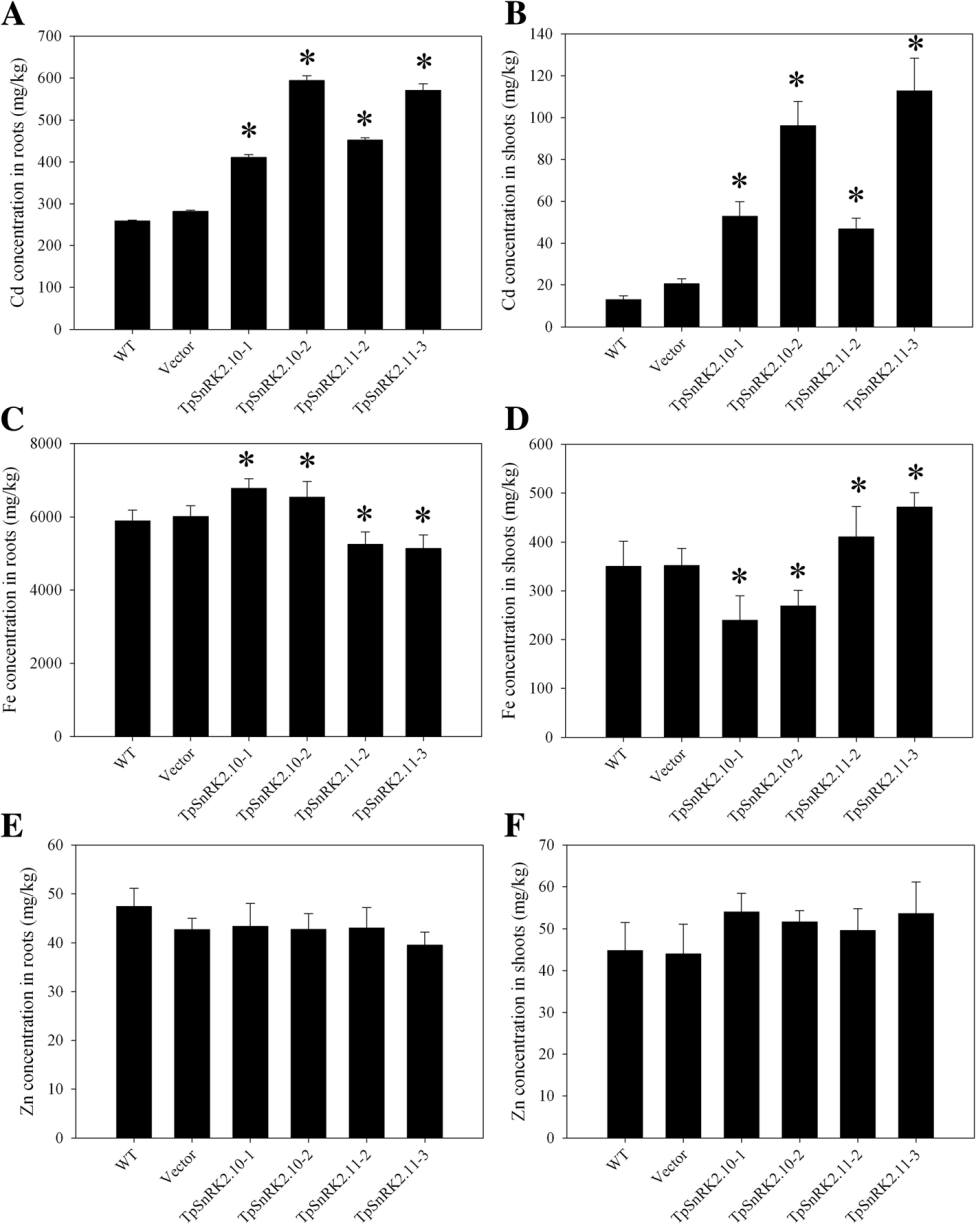
Metal concentrations in roots and shoots of *TpSnRK2.10*- and *TpSnRK2.11*-overexpressing *Arabidopsis*. **a-b** Cd concentration in roots (**a**) and shoots (**b**); **c-d** Fe concentration in roots (**c**) and shoots (**d**); **e-f** Zn concentration in roots (**e**) and shoots (**f**). Asterisk indicated significant difference when compared with WT at *P* < 0.05; values were means ± standard deviation (three biological replicates)

**Fig. 7:**
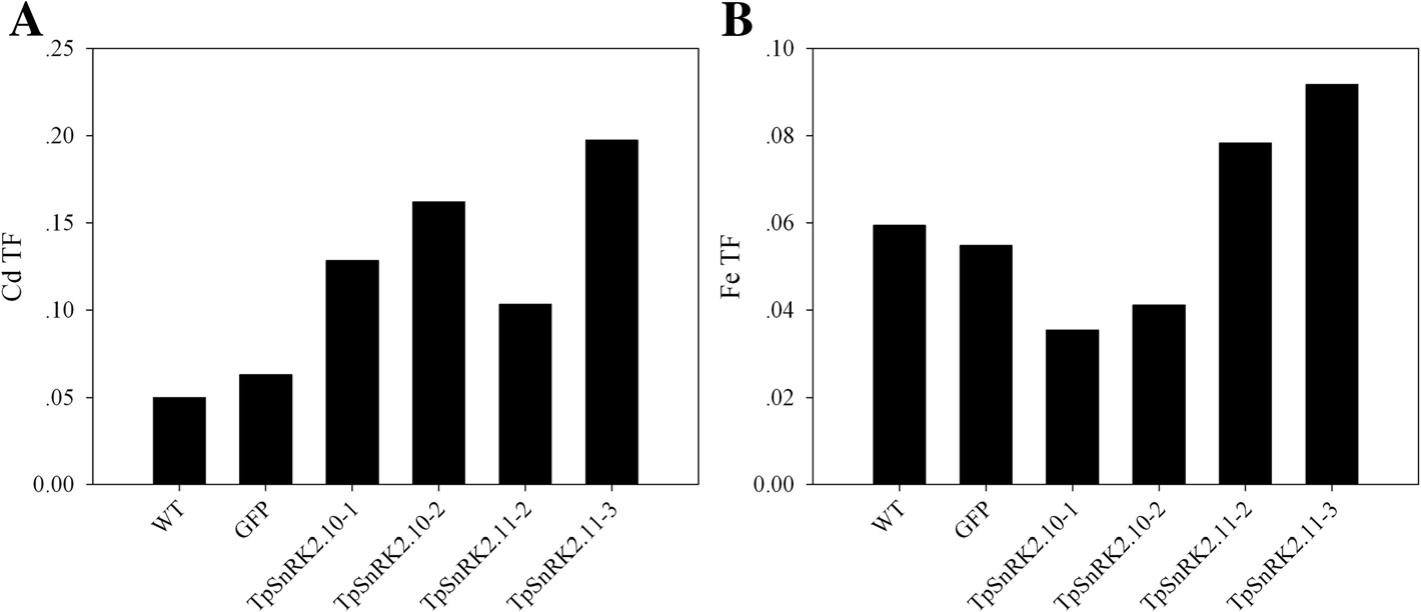
Cd and Fe translocation factors (TFs, the shoot-to-root Cd or Fe concentration ratio) of *TpSnRK2.10*- and *TpSnRK2.11*-overexpressing *Arabidopsis*. **a** Cd TF; B: Fe TF

Meanwhile, we analyzed Cd, Fe and Zn concentrations in roots and shoots grown in soil. Under 40 mg/kg CdCl_2_ stress, overexpression of *TpSnRK2.10* or *TpSnRK2.11* significantly increased Cd concentration in roots and shoots when compared with WT and vector line (Fig. 6a and b). Under none metal stress, overexpression of *TpSnRK2.10* significantly enhanced Fe concentration in roots (Fig. 6c), but reduced in shoots when compared with WT and vector line (Fig. 6d); in contrast, overexpression of *TpSnRK2.11* significantly reduced Fe concentration in roots (Fig. 6c), but enhanced in shoots when compared with WT and vector line (Fig. 6d). However, overexpression of *TpSnRK2.10* or *TpSnRK2.11* did not change Zn concentration in roots and shoots (Fig. 6e and f). Compared with WT, overexpression of *TpSnRK2.10* or *TpSnRK2.11* significantly enhanced Cd translocation factor (TF, the shoot-to-root Cd concentration ratio) (Fig. 7a); expression of *TpSnRK2.11* enhanced Fe TF, while overexpression of *TpSnRK2.10* reduced that (Fig. 7b). These results indicated that TpSnRK2.10 and TpSnRK2.11 mediate Cd and Fe uptake and translocation.

**Fig. 8:**
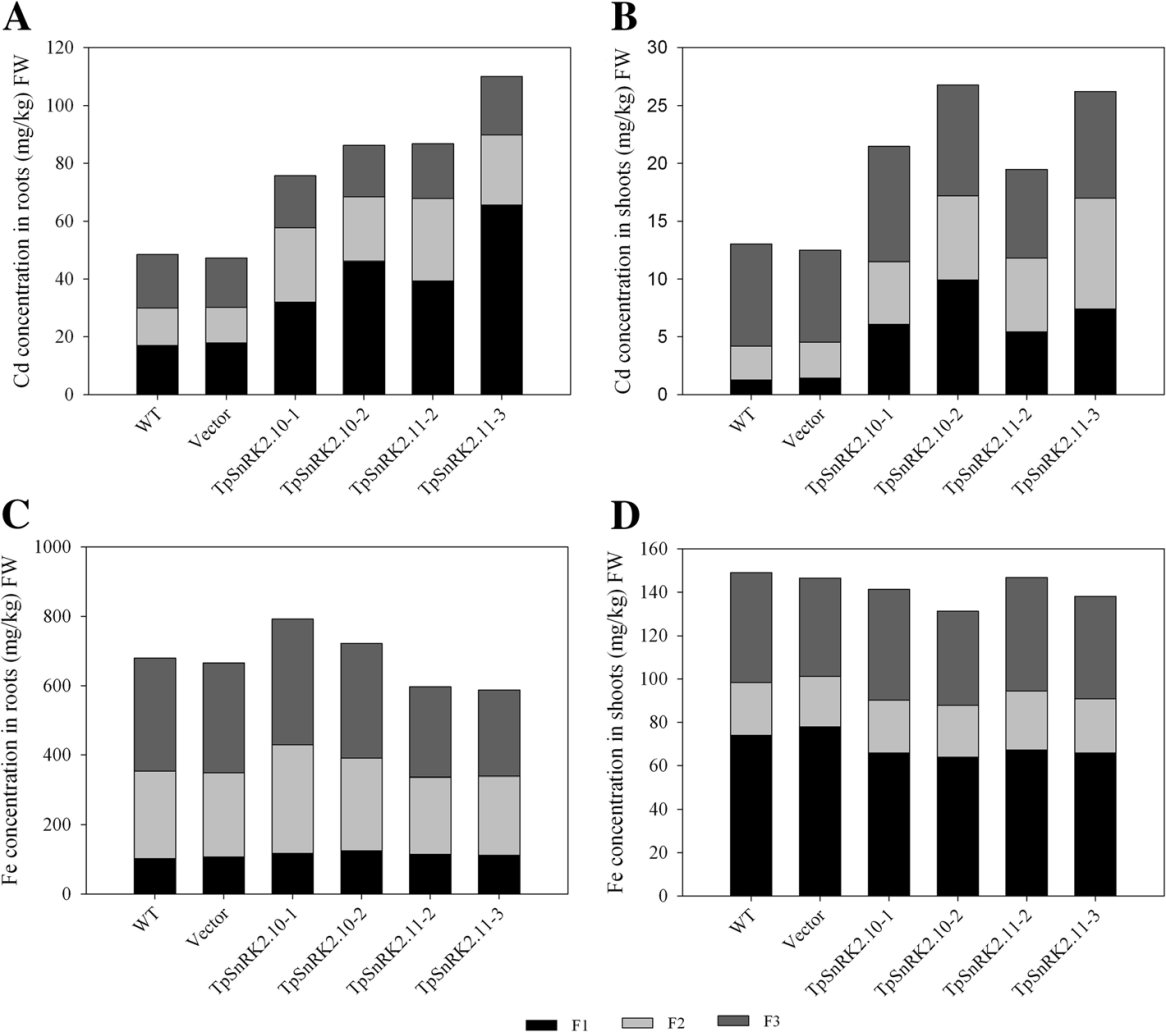
Subcellular distribution of Cd and Fe in roots and shoots of *TpSnRK2.10*- and *TpSnRK2.11*-overexpressing *Arabidopsis*. **a-b** Cd concentration in roots (A) and shoots (B); **c-d** Fe concentration in roots (C) and shoots (D). All values were means derived from three biological replicates. F1 represents cell wall fractions; F2 represents organelle fractions; F3 represents soluble fractions

To understand the physiological mechanisms that TpSnRK2.10 and TpSnRK2.11 participate in Cd and Fe uptake and translocation, we analyzed Cd and Fe subcellular distribution in overexpressing lines, WT and vector line. Compared with WT and vector line, overexpression of *TpSnRK2.10* and *TpSnRK2.11* dramatically increased Cd concentration of cell wall fraction and organelle fraction in roots and shoots (Fig. 8a and b). Overexpression of *TpSnRK2.10* increased Fe concentration of cell wall fraction and organelle fraction in roots (Fig. 8c), but decreased that of cell wall fraction in shoots when compared with WT and vector line (Fig. 8d). However, overexpression of *TpSnRK2.11* reduced Fe concentration in soluble fraction in roots (Fig. 8c), and cell wall fraction in shoots (Fig. 8d). These results implied that TpSnRK2.10 and TpSnRK2.11 probably influence Cd and Fe uptake and translocation via changing Cd and Fe subcellular distribution in roots and shoots.

**Fig. 9:**
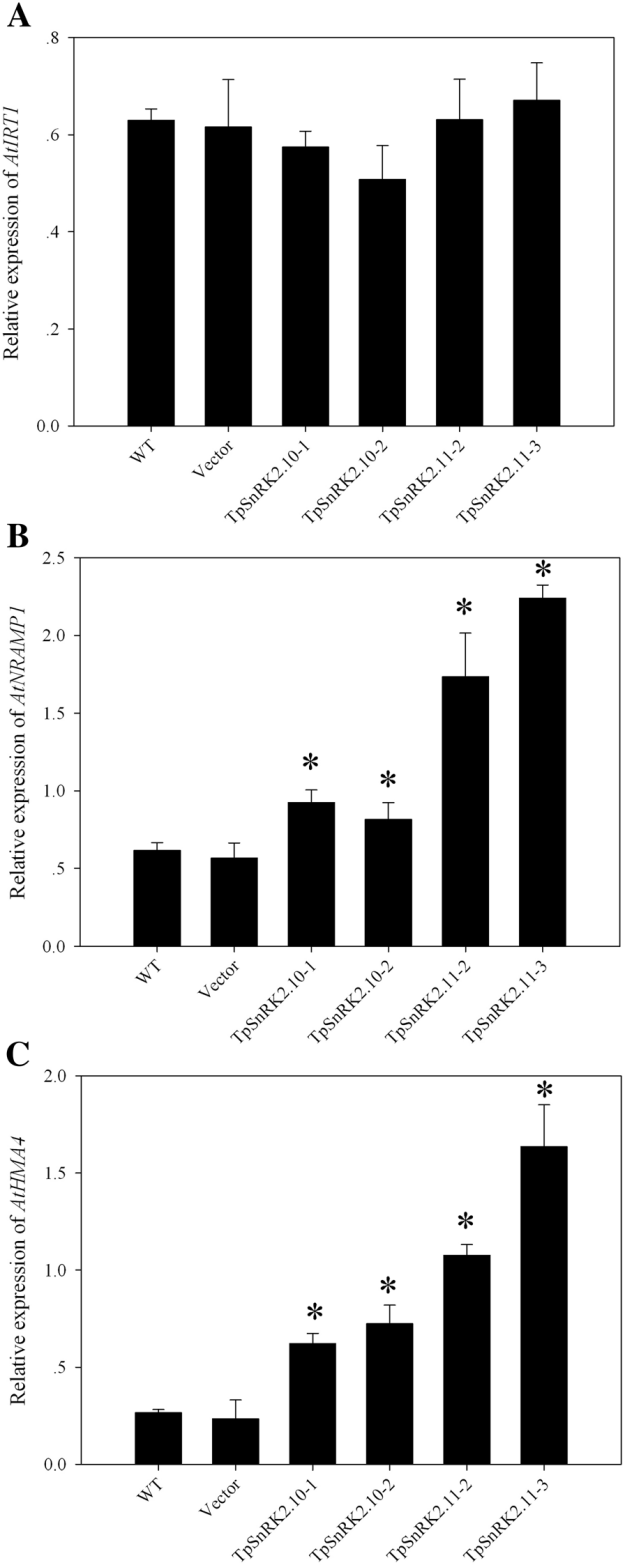
Relative expression of *AtIRT1*, *AtNRAMP1* and *AtHMA4* in transgenic lines. **a** Relative expression of *AtIRT1*; **b** relative expression of *AtNRAMP1*; **c** relative expression of *AtHMA4*. Samples were collected from shoot of Arabidopsis without metal stress. Asterisk indicated significant different when compared with WT at P < 0.05; value was mean ± standard deviation (three biological replicates)

Additionally, we further analyzed the expression of *AtIRT1*, *AtNRAMP1* and *AtHMA4* in overexpressing lines to understand whether *TpSnRK2.10* and *TpSnRK2.11* change their expression. Interestingly, we found that overexpression of *TpSnRK2.10* and *TpSnRK2.11* did not influence the expression of *AtIRT1* (Fig. 9a), but significantly enhanced the expression of *AtNRAMP1* (Fig. 9b) and *AtHMA4* (Fig. 9c).

## Discussion

In *Arabidopsis*, Cd can activate *AtSnRK2.4* and *AtSnRK2.10*; meanwhile, knockout of *AtSnRK2.4* enhances Cd tolerance [6]. Overexpression of wheat genes *TaSnRK2.3*, *TaSnRK2.4*, and *TaSnRK2.7* or *TaSnRK2.8* in *Arabidopsis* noticeably enhances drought, salinity and cold tolerance [18, 35–37]. Meanwhile, *SnRK2s* from tetraploid wheat are differently regulated by several abiotic stresses including drought, salt, ABA and cold [23, 24]. However, the roles of these SnRK2s from wheat in heavy metal responses and transport are still elusive. In this study, expression of *TpSnRK2.11* restored the growth of Cd sensitive strain; while expression of *TpSnRK2.10* increased the growth inhibition of Cd. The results suggested that TpSnRK2.10 and TpSnRK2.11 play different roles in Cd tolerance in yeast. Overexpression of *TpSnRK2.10* or *TpSnRK2.11*, however, did not change Cd tolerance in *Arabidopsis*, which was concluded from similar root growth and length of seedling under 40 μM CdCl_2_, and root and shoot dried weight of maturation under 40 mg/kg CdCl_2_. The discrepant results might result from the different genome buffering between yeast and *Arabidopsis*. Meanwhile, these results implied that the functions of TpSnRK2.10 and TpSnRK2.11 differ from that of AtSnRK2.4, which mediates Cd tolerance [6]. These differences might result from: (1) the low identity (68.6%) between TpSnRK2.11 and AtSnRK2.4, which includes different residues in several crucial motifs (ATP binding site, activation loop, and serine/threonine protein kinases activity site); (2) the different subcellular localizations that TpSnRK2.11 was localized to the cytoplasm, but AtSnRK2.4 was localized to the cytoplasm and nucleus [6]; and (3) the different responses to Cd, such as the expression of *TpSnRK2.11* was down-regulated by Cd, and the expression of *AtSnRK2.4* was up-regulated by Cd [6].

Many studies indicate that SnRK2 mediates the regulation of sucrose metabolism, phytochelatins synthesis, and sulfur metabolism [6, 11, 38], which play crucial roles in heavy metal binding, transport and tolerance [16, 19, 39]. Meanwhile, SnRK2s also regulate the expression of some genes that encode metal transporters and metal chelation, such as IRT and plant metallothionein [6, 12, 40]. Thus, SnRK2s would be involved in heavy metal uptake, translocation and tolerance. In *Arabidopsis*, knockout of *AtSnRK2.2* and *AtSnRK2.3* reduces Cd concentration in roots but increases that in shoots [12]; however, knockout of *AtSnRK2.4* did not affect Cd concentration, although it enhanced Cd tolerance [6]. In this study, overexpression of *TpSnRK2.10* or *TpSnRK2.11* significantly increased Cd concentration in roots and shoots, and enhanced Cd translocation. These results indicated that TpSnRK2.10 and TpSnRK2.11 are involved in Cd uptake and translocation in overexpressing *Arabidopsis*, which are different from the functions of AtSnRK2.2 and AtSnRK2.3 [12], and AtSnRK2.4 [6]. In *Arabidopsis*, AtIRT1 and AtNRAMP1 are major transporters for Cd uptake [13, 41]; their expression could be potentially mediated by SnRK2s through phosphorylation of ABFs [12]. In this study, overexpression of *TpSnRK2.10* or *TpSnRK2.11* in *Arabidopsis* enhanced the expression of *AtNRAMP1* to increase Cd uptake. Meanwhile, overexpression of *TpSnRK2.10* or *TpSnRK2.11* in *Arabidopsis* also enhanced the expression of *AtHMA4* that is responsible for Cd translocation in *Arabidopsis* [42], and enhanced Cd translocation from root-to-shoot.

Except transportation of Cd, AtNRAMP1 also transport Fe for its uptake [41]. Thus, overexpression of *TpSnRK2.10* or *TpSnRK2.11* in *Arabidopsis* enhanced Fe uptake and translocation. However, overexpression of *TpSnRK2.10* enhanced Fe concentration in roots, but reduced that in shoots, which implied that root retained more Fe to reduce Fe translocation. Conversely, overexpression of *TpSnRK2.11* reduced Fe concentration in roots, but increased that in shoots, which indicated that more Fe was transported into shoots. Thus, overexpression of *TpSnRK2.10* and *TpSnRK2.11* would differentially regulate other metal transporters that specifically transport Fe to mediate Fe translocation. For example, OsYSL15 only transports Fe for Fe uptake and translocation in rice [43]; HvYS1 specifically transports Fe for Fe uptake in barely [44]; and AtNRAMP3 pumps Fe from the vacuoles to the cytosol [45]. The different influences in Fe uptake and/or translocation implied that TpSnRK2.10 and TpSnRK2.11 have different biological functions, such as their different expression patterns.

Knockout of *AtSnRK2.4* reduced the synthesis of PCs [6], and overexpression of *AtSnRK2.6* increased the content of hemicellolosic polysaccharides of cell wall [11]. In plant, PCs chelate metals and then sequestrated into the vacuoles [16]; hemicellolosic polysaccharides chelate metals and binds in cell wall [19]. They affect metal binding and mobilization. In this study, overexpression of *TpSnRK2.10* and *TpSnRK2.11* might enhance the content of PCs and hemicellolosic polysaccharides. Thus, higher Cd concentration in cell wall fraction of roots and shoots was observed in overexpressing lines. The results implied that overexpression of *TpSnRK2.10* and *TpSnRK2.11* enhanced the require capacity of Cd in roots and shoots, resulting in the increased Cd concentration did not influence the growth. Meanwhile, increased Fe concentrations in cell wall and organelle fractions of roots were observed in *TpSnRK2.10*-overexpressing lines. Conversely, reduced Fe concentrations in soluble fraction of roots and cell wall fraction of shoots were detected in *TpSnRK2.11*-overexpressing lines. The changes of Fe concentration in roots and shoots were consistent with Fe translocation from roots to shoots. Thus, these results confirmed that TpSnRK2.10 and TpSnRK2.11 are involved in Cd and Fe distribution.

## Conclusion

In summary, our results indicate that TpSnRK2.10 and TpSnRK2.11 are involved in Cd and Fe accumulation and distribution in overexpressing lines, possibly by regulating *AtNRAMP1* and *AtHMA4* who have ABF or ABFs element in their promoters, and/or regulating the synthesis of phytochelatins and hemicellolosic polysaccharides. Since overexpression of *TpSnRK2.10* or *TpSnRK2.11* enhances Cd uptake and translocation from roots to shoots, knockout or knockdown of these two genes could help reduce Cd entrance into plant; or overexpression of these two genes could be used to phytoextract Cd from polluted soils.

## Additional file

Additional file 1: **Figure S1.** Relative expression of *TpSnRK2.10* (A) and *TpSnRK2.11* (B) in transgenic *Arabidopsis*. (DOCX 89 kb)

## Abbreviations

ABA: abscisic acid
ABFs: ABRE binding factors
ABRE: ABA response element
Cd: cadmium
CK: control
DPW: dwarf Polish wheat
Fe: ferrum
GFP: green fluorescent protein
IRT1: IRON-REGULATED TRANSPORTER1
SD: synthetic defined
SnRK2: sucrose nonfermenting-1 (SNF1)-related protein kinase 2
WT: wild type

## References

[1] Perrier F, Yan B, Candaudap F, Pokrovsky OS, Gourdain E, Meleard B (2016). Variability in grain cadmium concentration among durum wheat cultivars: impact of aboveground biomass partitioning. Plant Soil.

[2] DalCorso G, Farinati S, Furini A (2010). Regulatory networks of cadmium stress in plants. Plant Signal Behav.

[3] Yang Y, Xiong J, Chen R, Fu G, Chen T, Tao L (2016). Excessive nitrate enhances cadmium (cd) uptake by up-regulating the expression of *OsIRT1* in rice (*Oryza sativa*). Environ Exp Bot.

[4] Wang Y, Wang C, Liu Y, Yu K, Zhou Y (2018). GmHMA3 sequesters cd to the root endoplasmic reticulum to limit translocation to the stems in soybean. Plant Sci.

[5] Farinati S, DalCorso G, Varotto S, Furini A (2010). The *Brassica juncea* BjCdR15, an ortholog of *Arabidopsis* TGA3, is a regulator of cadmium uptake, transport and accumulation in shoots and confers cadmium tolerance in transgenic plants. New Phytol.

[6] Kulik A, Anielska-Mazur A, Bucholc M, Koen E, Szymańska K, Żmieńko A (2012). SNF1-related protein kinases type2 are involved in plant responses to cadmium stress. Plant Physiol.

[7] Cheng Y, Wang C, Chai S, Shuai W, Sha L, Zhang H (2018). Ammonium N influences the uptakes, translocations, subcellular distributions and chemical forms of cd and Zn to mediate the cd/Zn interactions in dwarf polish wheat (*Triticum polonicum* L.) seedlings. Chemosphere..

[8] Yeh CM, Chien PS, Huang HJ (2007). Distinct signaling pathways for induction of MAP kinase activities by cadmium and copper in rice roots. J Exp Bot.

[9] Liu X, Kim KE, Kim K, Nguyen XC, Han HJ, Jung MS (2010). Cadmium activates *Arabidopsis* MPK3 and MPK6 via accumulation of reactive oxygen species. Phytochemistry..

[10] Fujii H, Verslues PE, Zhu JK (2007). Identification of two protein kinases required for abscisic acid regulation of seed germination, root growth, and gene expression in *Arabidopsis*. Plant Cell.

[11] Zheng Z, Xu X, Crosley RA, Greenwalt SA (2010). SunY, Blakeslee B, et al. the protein kinase SnRK2.6 mediates the regulation of sucrose metabolism and plant growth in *Arabidopsis*. Plant Physiol.

[12] Fan SK, Fang XZ, Guan MY, Ye YQ, Lin XY, Du ST (2014). Exogenous abscisic acid application decreases cadmium accumulation in *Arabidopsis* plants, which is associated with the inhibition of IRT1-mediated cadmium uptake. Front Plant Sci.

[13] Vert G, Grotz N, Dédaldéchamp F, Gaymard F, Guerinot ML, Briat JF (2002). IRT1, an *Arabidopsis* transporter essential for iron uptake from the soil and plant growth. Plant Cell.

[14] Thomine S, Wang R, Ward JM, Crawford NM, Schroeder JI (2000). Cadmium and iron transport by members of a plant metal transporter family in *Arabidopsis* with homology to *Nramp* genes. P Natl Acad Sci USA.

[15] Cailliatte R, Lapeyre B, Briat JF, Mari S, Curie C (2009). The NRAMP6 metal transporter contributes to cadmium toxicity. Biochem J.

[16] Brunetti P, Zanella L, De Paolis A, Di Litta D, Cecchetti V, Falasca G (2015). Cadmium-inducible expression of the ABC-type transporter *AtABCC3* increases phytochelatin-mediated cadmium tolerance in *Arabidopsis*. J Exp Bot.

[17] Yoshida R, Umezawa T, Mizoguchi T, Takahashi S, Takahashi F, Shinozaki K (2006). The regulatory domain of SRK2E/OST1/SnRK2.6 interacts with ABI1 and integrates abscisic acid (ABA) and osmotic stress signals controlling stomatal closure in *Arabidopsis*. J Biol Chem.

[18] Zhang H, Mao X, Wang C, Jing R. Overexpression of a common wheat gene *TaSnRK2.8* enhances tolerance to drought, salt and low temperature in *Arabidopsis*. PloS One. 2010;5(12):e16041. 10.1371/journal.pone.0016041 PMC3012728 21209856

[19] Song XQ, Liu LF, Jiang YJ, Zhang BC, Gao YP, Liu XL (2013). Disruption of secondary wall cellulose biosynthesis alters cadmium translocation and tolerance in rice plant. Mol Plant.

[20] Johnson RR, Wagner RL, Verhey SD, Walke-Simmons MK (2002). The abscisic acid-responsive kinase PKABA1 interacts with a seed-specific abscisic acid response element-binding factor, TaABF, and phosphorylates TaABF peptide sequences. Plant Physiol.

[21] Kobayashi Y, Murata M, Minami H, Yamamoto S, Kagaya Y, Hobo T (2005). Abscisic acid-activated SNRK2 protein kinases function in the gene-regulation pathway of ABA signal transduction by phosphorylating ABA response element-binding factors. Plant J.

[22] Zhang H, Li W, Mao X, Jing R, Jia H (2016). Differential activation of the wheat SnRK2 family by abiotic stresses. Front Plant Sci.

[23] Wang Y, Wang X, Gu M, Kang H, Zeng J, Fan X (2015). Cloning and characterization of four novel *SnRK2* genes from *Triticum polonicum*. Biol Plantarum.

[24] Jiang Y, Wang Y, Huang Z, Kang H, Sha L, Fan X (2017). Cloning and characterization of four *TpSnRK2s* from dwarf polish wheat. Biol Plantarum.

[25] Wang X, Wang C, Sheng H, Wang Y, Zeng J, Kang H (2017). Transcriptome-wide identification and expression analysis of ABC transporters in dwarf polish wheat under metal stresses. Biol Plantarum..

[26] The International Wheat Genome Sequencing Consortium (2014). A chromosome-based draft sequence of the hexaploid bread wheat (*Triticum aestivum*) genome. Science..

[27] Peng F, Wang C, Zhu J, Zeng J, Kang H, Fan X (2018). Expression of *TpNRAMP5*, a metal transporter from polish wheat (*Triticum polonicum* L.), enhances the accumulation of cd, co and Mn in transgenic *Arabidopsis* plants. Planta..

[28] Yoo SD, Cho YH, Sheen J (2007). Arabidopsis mesophyll protoplasts: a versatile cell system for transient gene expression analysis. Nat Protoc.

[29] Clough SJ, Bent AF (1998). Floral dip: a simplified method for *Agrobacterium*-mediated transformation of *Arabidopsis thaliana*. Plant J.

[30] Ihnatowicz A, Siwinska J, Meharg AA, Carey M, Koornneef M, Reymond M (2014). Conserved histidine of metal transporter AtNRAMP1 is crucial for optimal plant growth under manganese deficiency at chilling. New Phytol.

[31] Boonyaves K, Gruissem W, Bhullar NK (2016). NOD promoter-controlled *AtIRT1* expression functions synergistically with *NAS* and *FERRITIN* genes to increase iron in rice grains. Plant Mol Biol.

[32] Chen ZR, Kuang L, Gao YQ, Wang YL, Salt DE, Chao DY (2018). AtHMA4 drives natural variation in leaf Zn concentration of *Arabidopsis thaliana*. Front Plant Sci.

[33] Giniger E, Varnum SM, Ptashne M (1985). Specific DNA binding of GAL4, a positive regulatory protein of yeast. Cell..

[34] Wang Y, Yu KF, Poysa V, Shi C, Zhou YH (2012). A single point mutation in GmHMA3 affects cadmium (cd) translocation and accumulation in soybean seeds. Mol Plant.

[35] Mao X, Zhang H, Tian S, Chang X, Jing R (2010). TaSnRK2.4, an SNF1-type serine/threonine protein kinase of wheat (*Triticum aestivum* L.), confers enhanced multistress tolerance in *Arabidopsis*. J Exp Bot.

[36] Zhang H, Mao X, Jing R, Chang X, Xie H (2011). Characterization of a common wheat (*Triticum aestivum* L.) *TaSnRK2.7* gene involved in abiotic stress responses. J Exp Bot.

[37] Tian S, Mao X, Zhang H, Cheng S, Zhai C, Yang S (2013). Cloning and characterization of *TaSnRK2.3*, a novel SnRK2 gene in common wheat. J Exp Bot.

[38] González-Ballester D, Pollock SV, Pootakham W, Grossman AR (2008). The central role of a SNRK2 kinase in sulfur deprivation responses. Plant Physiol.

[39] Liang T, Ding H, Wang G, Kang J, Pang H, Lv J (2016). Sulfur decreases cadmium translocation and enhances cadmium tolerance by promoting sulfur assimilation and glutathione metabolism in *Brassica chinensis* L. Ecotox Environ Safe.

[40] Nakashima K, Fujita Y, Kanamori N, Katagiri T, Umezawa T, Kidokoro S (2009). Three Arabidopsis SnRK2 protein kinases, SRK2D/SnRK2.2, SRK2E/SnRK2.6/OST1 and SRK2I.SnRK2.3, involved in ABA signaling are essential for the control of seed development and dormancy. Plant Cell Physiol.

[41] Cailliatte R, Schikora A, Briat JF, Mari S, Curie C (2010). High-affinity manganese uptake by the metal transporter NRAMP1 is essential for *Arabidopsis* growth in low manganese conditions. Plant Cell.

[42] Wong CKE, Cobbett CS (2009). HMA P-type ATPases are the major mechanism for root-to-shoot cd translocation in *Arabidopsis thaliana*. New Phytol.

[43] Lee S, Chiecko JC, Kim SA, Walker EL, Lee Y, Guerinot ML (2009). Disruption of OsYSL15 leads to iron inefficiency in rice plants. Plant Physiol.

[44] Murata Y, Ma JF, Yamaji N, Ueno D, Nomoto K, Iwashita T (2006). A specific transporter for iron (III)-phytosiderophore in barely roots. Plant J.

[45] Thomine S, Lelievre F, Debarbieux E, Schroeder JI, Barbier-Brygoo H (2003). AtNRAMP3, a multispecific vacuolar metal transporter involved in plant responses to iron deficiency. Plant J.

