# Supplementary material for: The polish wheat (Triticum polonicum L.) TpSnRK2.10 and TpSnRK2.11 meditate the accumulation and the distribution of cd and Fe in transgenic Arabidopsis plants
Source: BMC Genomics. 2019 Mar 12;20:210. doi: 10.1186/s12864-019-5589-1 (PMC6417267; doi:10.1186/s12864-019-5589-1)
Supplement: Supplementary file 1 — Figure S1. Relative expression of TpSnRK2.10 (A) and TpSnRK2.11 (B) in transgenic Arabidopsis. (DOCX 89 kb) [file 12864_2019_5589_MOESM1_ESM.docx]

**
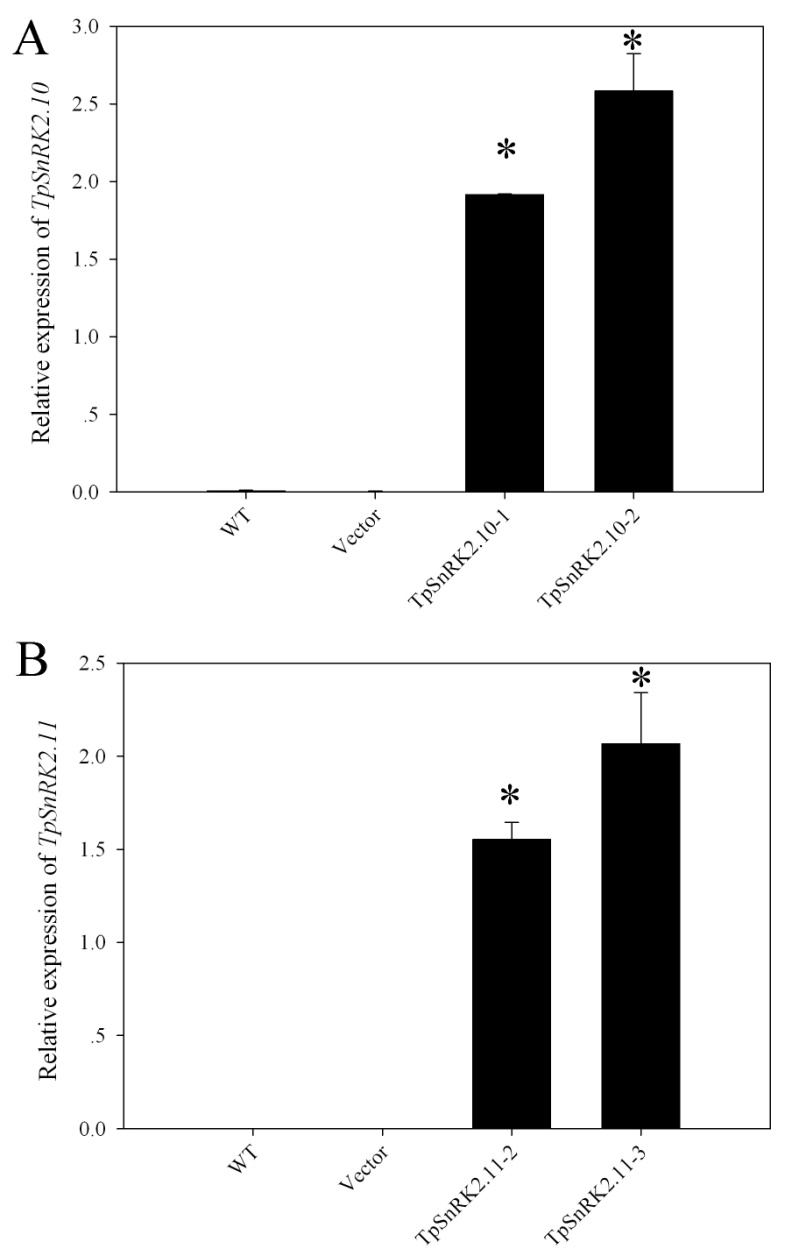
**

**Additional file 1: Figure S1.** Relative expression of *TpSnRK2.10* (A) and *TpSnRK2.11* (B) in transgenic *Arabidopsis*. Asterisk indicated significant different when compared with WT at P < 0.05; value was mean ± standard deviation (three biological replicates).
